# Increased prevalence of *Borrelia burgdorferi *infections in Bernese Mountain Dogs: a possible breed predisposition

**DOI:** 10.1186/1746-6148-3-15

**Published:** 2007-07-12

**Authors:** Bernhard Gerber, Simone Eichenberger, Max M Wittenbrink, Claudia E Reusch

**Affiliations:** 1 Clinic for Small Animal Internal Medicine, Vetsuisse Faculty University of Zurich, Winterthurstrasse 260, 8057 Zurich, Switzerland; 2 Institute of Veterinary Bacteriology, Vetsuisse Faculty University of Zurich, Winterthurstrasse 260, 8057 Zurich, Switzerland

## Abstract

**Background:**

Glomerulonephritis in dogs has been associated with *B. burgdorferi *infections. In Bernese Mountain Dogs with glomerulonephritis antibodies against *B. burgdorferi *have been found in most dogs, raising the question if the breed is predisposed to infections with *B. burgdorferi*. The aim of this study was to determine the prevalence of antibodies against *B. burgdorferi sensu lato *in a well defined population of Bernese Mountain Dogs and to compare this prevalence with data from dogs of other breeds.

**Results:**

160 Bernese Mountain Dogs and 62 control dogs (large breed dogs with long hair) were included. All dogs were considered healthy according to a questionnaire filled out by the owner, complete blood count, chemistry panel, urinalysis and urine culture. Bernese Mountain Dogs and control dogs were kept in similar environments. Seroprevalence of *B. burgdorferi *was assessed by ELISA and Western blot and was 58% in Bernese Mountain Dogs compared to 15% in control dogs. This difference was significant. Neither antibodies against leptospires nor vaccination or hair coat color influenced the results.

**Conclusion:**

The cause of the considerably higher prevalence of antibodies against *B. burgdorferi *in Bernese Mountain Dogs and it's consequences are not known. A breed predisposition can be suspected.

## Background

Glomerulonephritis in dogs has been associated with *B. burgdorferi *infections [1-5] and in some studies spirochetes were detected in the kidneys [2,3] and the urine [2]. However some of the authors questioned the relationship of a renal lesion with *B. burgdorferi *[1,3]; still others assumed *B. burgdorferi *to be the causative agent for renal lesions [2]. In Bernese Mountain Dogs, a familial glomerulonephritis was reported [4,5]. However, antibodies against *B. burgdorferi *were found in most dogs, raising the question of whether the occurrence of glomerular disease in Bernese Mountain Dogs is related to an infection with *B. burgdorferi *or if the breed is predisposed to infections with *B. burgdorferi*.

The aim of this study was to determine the prevalence of antibodies against *B. burgdorferi sensu lato *in a well defined population of Bernese Mountain Dogs and to compare this prevalence with data from dogs of other breeds from a similar environment.

## Results

### Dogs

One hundred and sixty Bernese Mountain Dogs and 62 control dogs were included in the study. Age, gender, hair coat color and breed are depicted in Table 1. Bernese Mountain Dogs were significantly younger than the control dogs (p = 0.01). Gender distribution was the same in both groups (p = 0.41). Fifty-six of the 62 control dogs belonged to 8 different long haired large breeds. The remaining 6 dogs were mixed-breed dogs with Collie, German Shepherd and Flat-Coated Retriever as dominant breeds.

**Table 1:** Breed, age, gender and hair coat color of dogs included in the study

Breed	number of dogs	**Age^1 ^[years]**		**Gender [number of dogs]**				hair coat color
			
		Range	Median	f	fs	m	mn	
**Bernese Mountain Dogs**	**160**	**1–11**	**4**	**94**	**21**	**35**	**10**	**dark**

Landseer	28	1–12	5	16	2	8	2	fair
Newfoundland	12	3–8	6	9	1	2	0	dark
Flat-coated Retriever	8	1–7	1	4	0	3	1	dark
Golden Retriever	3	1–5	4	3	0	0	**0**	fair
Saint Bernard	2	2/5	3.5	1	1	0	0	fair
Belgian Sheppard	1	2	2	1	0	0	0	fair
Mastin de los Pirineos	1	11	11	0	0	1	0	fair
Tibetan Mastiff	1	5	5	0	0	1	0	fair
Mixed breed dogs	6	2–8	6.5	0	1	1	4	5 fair, 1 dark
Control dogs total	**62**	**1–12**	**5**	**34**	**5**	**16**	**7**	**41 fair, 21 dark**

f = female, fs = female spayed, m = male, mk = male neutered

^1^Significant difference between Bernese Mountain Dogs and control dogs (p = 0.01).

**Figure 1:**
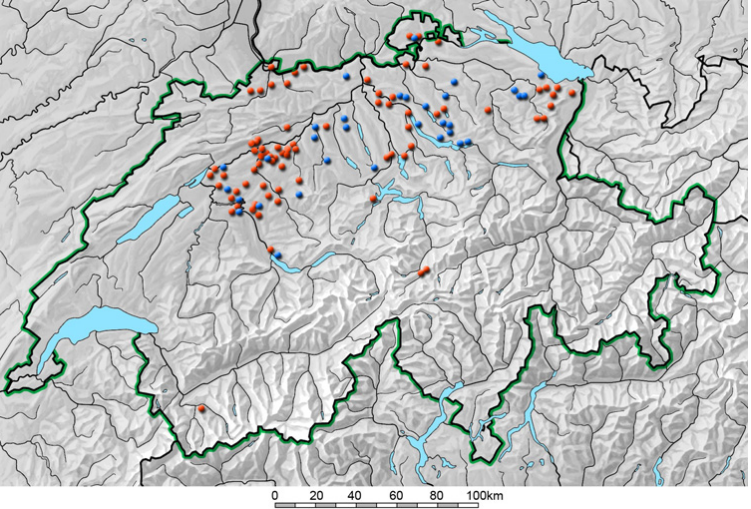
**Map of Switzerland with the geographical distribution of tested dogs**. Origin of Bernese Mountain Dogs (red dots) and control dogs (blue dots).

The geographical distribution of the places where the dogs lived is depicted in (Figure 1).

The evaluation of the replies given to the questionnaires are depicted in Table 2. Analysis of the answers only revealed significant differences between the groups for the frequency of attached ticks. Significantly more Bernese Mountain Dog owners (44%) answered yes to the question whether the dogs often had attached ticks compared to owners of control dogs (25%; p = 0.01). The significance disappeared if only dark haired control dogs (n = 20) were compared with Bernese Mountain Dogs even though the percentage remained the same (25% and 44% respectively; p= 0.08).

**Table 2:** Evaluation of replies to questions regarding health status of the dogs by questionnaire

		**Bernese Mountain Dogs**			**Control dogs**				
		
		*B. burgdorferi *serology		total	*B. burgdorferi *serology				total
						
		positive	negative		positive		negative		
						
					hair color		hair color		
						
					fair	dark	fair	dark	
Does your dog often have attached ticks?^1^	Yes	40	18	58	1	2	7	3	13
	No	40	33	73	1	4	24	11	40
Do you perform tick prevention?	Yes	52	38	90	2	6	23	5	36
	No	26	14	40	0	0	7	7	14
Did your dog suffer from infectious diseases?	No	62	47	109	2	3	25	12	42
	Yes	15	4	19	0	3	5	2	10
General health	Normal	80	52	132	2	6	30	14	52
	Abnormal	0	0	0	0	0	0	0	0
Endurance	Normal	76	50	126	2	6	28	14	50
	Decreased	4	2	6	0	0	2	0	2
Weight loss	No	75	52	127	2	6	30	14	52
	Yes	3	1	4	0	0	0	0	0
Skin normal	Yes	69	48	117	1	6	28	14	49
	No	9	4	13	1	0	2	0	3
Appetite	Normal	72	50	122	1	6	30	14	51
	Decreased	5	2	7	0	0	0	0	0
	Increased	1	0	1	1	0	0	0	1
Thirst	Normal	72	52	124	2	6	29	14	51
	Decreased	5	0	5	0	0	1	0	1
	Increased	1	0	1	0	0	0	0	0
Vomiting	No	77	52	129	2	6	29	14	51
	Yes	1	0	1	0	0	1	0	1
Coughing	No	77	52	129	2	6	30	14	52
	Yes	0	0	0	0	0	0	0	0
Urine volume	Normal	74	52	126	2	6	30	14	52
	Decreased	1	0	1	0	0	0	0	0
	Increased	1	0	1	0	0	0	0	0
Defecation	Normal	76	51	76	2	6	30	14	52
	Diarrhea	2	1	2	0	0	0	0	0
Lameness	No	46	36	46	1	6	24	14	45
	Yes	5	3	5	1	0	6	0	7
Fever	No	80	53	80	2	6	30	14	52
	Yes	0	0	0	0	0	0	0	0
Edema	No	51	39	51	2	6	30	14	52
	Yes	0	0	0	0	0	0	0	0

^1^Significant difference between Bernese Mountain Dogs and control dogs (p = 0.01).

The answers to the questions about the environment in which the dogs lived are depicted in Table 3. Significant differences were found between dogs which lived in a rural or a urban environment and for the percentage of time spent in the woods. A significantly larger number of Bernese Mountain Dogs (95%) lived in rural areas compared to control dogs (79%; p = 0.001). Looking at the two groups separately, living in rural areas did not lead to a higher prevalence in antibodies against *B. burgdorferi *compared to an urban environment. The reported percentage of time spent in the woods during walks was significantly higher in Bernese Mountain Dogs with antibodies against *B. burgdorferi *compared to those without them (p = 0.049). In control dogs no significant difference was found (p = 0.90).

**Table 3:** Evaluation of replies to questions asked by telephone interview regarding the environment the dogs lived in

		**Bernese Mountain Dogs**		**Control dogs**	
		
		number of dogs	*B. burgdorferi *positive [%]	number of dogs	*B. burgdorferi *positive [%]
Was the area you lived in rural or urban^1^	Rural	136	59	37	11
	Urban	6	50	10	30
Did your dogs have access to the house or did he live only in a kennel?	Access to house	127	56	41	17
	Kennel only	16	81	6	0
Had your dogs access to a run?	Yes	136	60	45	16
	No	7	43	2	0
Could your dog escape from the house or the run?	Yes	28	50	4	0
	No	115	61	43	16
How often did you walk the dog a day?	1 time	68	62	18	11
	2 times	51	57	16	19
	3 times	20	50	8	0
	>3 times	3	67	5	40
What percentage (%) of time did you spend in the woods on your walks?^2^	Range	0–100		0–100	
	Median	50		50	

^1^Significant difference between Bernese Mountain Dogs and control dogs (p = 0.001).

^2^Bernese Mountain Dogs with antibodies against *B. burgdorferi *spent a significantly higher percentage of time in the woods on walks than those without (p = 0.049).

### Antibodies against *B. burgdorferi*

**Figure 2:**
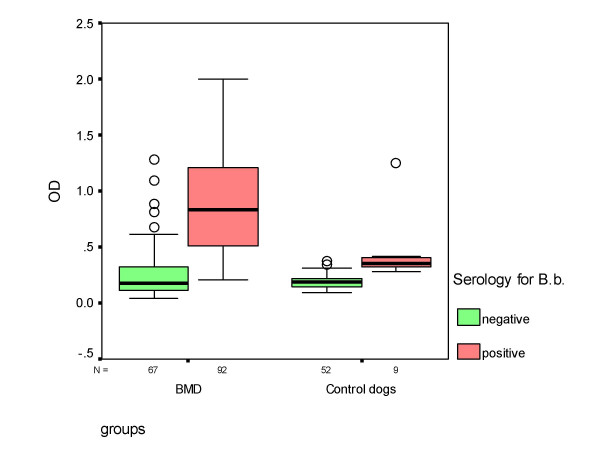
***B. burgdorferi *ELISA results (optical density 405 nm) from Bernese Mountain Dogs and control dogs**. OD = optical density, BMD = Bernese Mountain Dogs, B.b. = *Borrelia burgdorferi*

**Figure 3:**
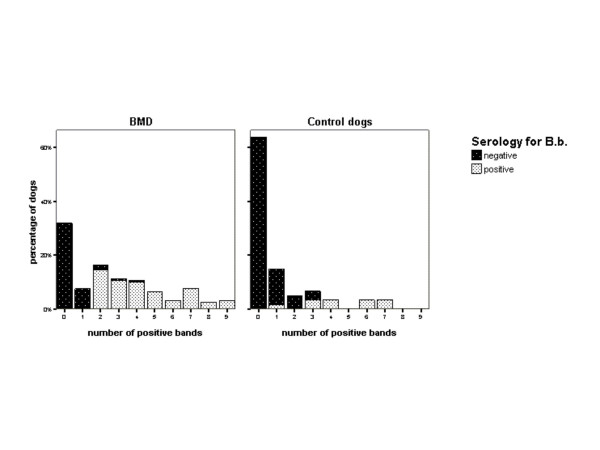
**Number of positive bands depicted in a Western blot for *B. burgdorferi *from serum of Bernese Mountain Dogs and control dogs**. BMD = Bernese Mountain Dogs, B.b. = *Borrelia burgdorferi*

In 160 Bernese Mountain Dogs and in 61 control dogs antibodies against *B. burgdorferi *were determined with both an ELISA and a Western blot. Of the Bernese Mountain Dogs, 92 (58%) had a positive ELISA with a positive Western blot, while in the control dogs this only happened in 9 (15%) dogs. This difference was significant (p < 0.001). In positive dogs ODs ranged from 0.21 to 2.00 (median 0.75) in negative dogs from 0.04 to 1.28 (median 0.18) (Figure 2). The ODs of positive Bernese Mountain Dogs were significantly higher than those of positive control dogs (p < 0.001). Seropositive dogs had 1 to 7 bands in the Western blot (median 4) while seronegative dogs had 0 to 3 bands (median 0) (Figure 3). The serology results are summarizes in Table 4. Control dogs with a dark coat had significantly more antibodies against *B. burgdorferi *(28%) when compared with control dogs with a fair coat (7%; p = 0.03). Bernese Mountain Dogs whose owners reported frequently attached ticks did not have antibodies against *B. burgdorferi *significantly more often (69%) compared to Bernese Mountain dogs whose owners reported infrequently attached ticks (55%; p = 0.07). Control dogs whose owners reported frequently attached ticks did not have antibodies against *B. burgdorferi *significantly more often than control dogs whose owners reported infrequently attached ticks (23% and 13% respectively; p = 0.30).

**Table 4:** Results of serologic testing for *B. burgdorferi*

	Bernese Mountain Dogs (n = 160)		**Control dogs (n = 62)**			
	
Serology*	number of dogs	%	number of dogs	%	coat color	
					
					**fair**	**dark**
positive^1,2^	92	58	9	15	3	6
negative	68	42	53	85	38	15
total	160	100	62	100	41	21

* ELISA and Western blot positive

^1^Significant difference between Bernese Mountain Dogs and control dogs (p < 0.001).

^2^Significant difference between control dogs with fair and dark coat color (p < 0.03).

### Comparison of antibodies against *B. burgdorferi *and against leptospires

Of 92 Bernese Mountain Dogs with antibodies against *B. burgdorferi*, 53 (58%) had antibodies against leptospires as well and of the 68 without antibodies against *B. burgdorferi*, 33 (49%) had antibodies against leptospires (p = 0.16). Of 9 control dogs with antibodies against *B. burgdorferi *there were 6 (67%) with antibodies against leptospires. and of 53 negative control dogs 28 (53%) had antibodies against leptospires (p = 0.35).

### Antibodies against *B. burgdorferi *and vaccination against Lyme borreliosis

Four Bernese Mountain dogs and 6 control dogs had been vaccinated against Lyme borreliosis. All 4 Bernese Mountain dogs had a positive ELISA but only 3 had a positive Western blot. Of the 6 control dogs, 3 had a positive ELISA and a positive Western blot and 3 were negative on both tests.

## Discussion

The higher prevalence of antibodies against *B. burgdorferi *in Bernese Mountain Dogs indicates a breed predisposition. Susceptibility in some breeds to a certain infection is known from other diseases. For instance Rottweiler, American Pit Bull Terrier, Doberman Pinscher, Pomeranian, and German Sheperd Dog are breeds at significantly greater risk for parvovirus enteritis than mixed breed dogs [6]. The reason is not known. However common ancestry has been associated with this. Intense breeding might have led to a decrease in defense against infections. This might also be true for Bernese Mountain Dogs, a breed that is known for intense breeding and that has a narrow gene pool. This is supported by the fact that several diseases are prevalent in Bernese Mountain Dogs such as bleeding tendency, epilepsy, and malignant histiocytosis [7-11]. However no infections have been described so far. In Cavalier King Charles Spaniels a breed that was known to be prone to *Pneumocystis *pneumonia infections, it was found that there was a immunoglobulin deficiency in the affected dogs indicating defect in immunity in these dogs [12]. The findings in the present study are unique as infections with *B. burgdorferi *are not causing disease. Furthermore, no immunodeficiency is known in Bernese Mountain Dogs. At the time, *B. burgdorferi *was associated with glomerulonephritis in Bernese Mountain Dog, no direct relation to the disease could be made. It is possible that the Bernese Mountain Dogs with glomerulonephritis in this study had antibodies against *B. burgdorferi *because the over all prevalence of antibodies was so high in this breed [5]. Labrador- and Golden Retrievers were found to be overrepresented in a group of dogs with distinctive renal lesions attributed to Lyme disease and also among seropositive dogs in a survey performed in Texas [1,13], indicating some breed predilection for *B. burgdorferi *infections. However Bernese Mountain Dogs were not mentioned in the studies.

It is well established that Borrelia organisms evade the immune system in different ways and host factors become more important the less pathogen the responsible organisms are [14,15]. In human patients with Lyme disease-associated erythema migrans, the carriage rate of leukocyte class II alleles DRB1*0101 and DRB1*0101-DQB1*0501 was higher in patients with the least pathogen *B. burgdorferi *genotype [15]. The immunologic event causing this association was not known but as DRB1 alleles are located close to certain major histocompatibility complex-encoded complement genes, it was speculated that variants of these complement genes might be in linkage disequilibrium with the DRB1 alleles [15,16]. The innate immune response plays an important role in the early response of Borrelia [17] but it also plays a role in the development of certain glomerular diseases. Dogs with a genetically determined deficiency of complement C3 more often develop renal and infectious diseases [18]. The occurrence of a complement disturbance would explain the co-occurrence of infection with *B. burgdorferi *and glomerular disease. However no such disturbance is known in Bernese Mountain Dogs so far.

In one study 5% to 34% of the *I. ricinus *ticks in Switzerland were infected with *B. burgdorferi *and infected ticks were found in all areas where ticks were collected [19]. These figures remained stable in later studies [20-25]. Even though owners of control dogs reported more often that they lived in urban areas, the Bernese Mountain Dogs and the control dogs in the present study were kept similar, lived in the same areas of Switzerland, were walked in the same frequency and for the equal amount of time in the woods. In addition all tick-infested areas in Switzerland harbor infected ticks. Based on this Bernese Mountain Dogs did not appear to have a higher risk of tick exposure compared to control dogs. Furthermore in a Dutch study, prevalence of antibodies were not different between dogs considered at high risk to a *B. burgdorferi *infection (hunting dogs) and those considered at low risk (pet dogs) [26]. One explanation for this was that the rate of outdoor walking in house dogs was considered higher and this also applies to Bernese Mountain Dogs or other large breed dogs in Switzerland. Nevertheless Bernese Mountain Dog owners in this study reported that their dogs had attached ticks more often than owners of control dogs and it is known that seropositivity among dogs is positively associated with increased tick exposure [27]. However the question whether the dogs had attached ticks more often was not specified and was therefore subject to the individual judgment of the owner. Furthermore neither the Bernese Mountain Dogs nor the control dogs which had a high frequency of attached ticks reported, had a significantly higher prevalence of antibodies against *B. burgdorferi *compared to those which did not frequently have ticks.

A possible reason for the increased exposure of Bernese Mountain Dogs was the dark hair coat. In dark hair it is more difficult for the owner to detect ticks than in dogs with fair hair color. This would allow more time for the Borrelia species to move from the tick to the host and infect the dogs [28-30]. In the control dogs it could be shown that the ones with dark hair had significantly more often antibodies than those with fair hair (28% versus 7%). However, if dark haired control dogs were compared with Bernese Mountain Dogs, it could be seen that they also had significantly less often antibodies against *B. burgdorferi *compared to Bernese Mountain Dogs even though statistically significant differences in reported tick exposure disappeared. This indicates that hair color is not the explanation for the higher seroprevalence of antibodies against *B. burgdorferi *in Bernese Mountain Dogs. Furthermore it was found that people with white clothing attracted more ticks than people in dark clothing in the same environment over the same time period, indicating that fair hair might even attract more ticks than dark hair [31].

Results of serologic tests are not consistent. The specificity of whole cell ELISAs is limited because of cross reactivity with other organisms [32]. Even though Western blot was performed for the confirmation of the ELISA results antibodies of leptospires were measured to rule out cross reaction in the ELISA. Antibodies against leptospires are known to cross-react with antibodies against *B. burgdorferi *and it was found that antibodies against leptospires are common in healthy dogs in Switzerland [33,34]. Results of the microscopic agglutination test (MAT) for antibodies against leptospires showed that there was no influence of leptospires on the results of antibody tests for *B. burgdorferi*. Vaccination can influence ELISA results [35]. In Switzerland dogs are rarely vaccinated against *B. burgdorferi *and in the present study only 10 dogs were vaccinated, which did not seem to influence the study results.

There are different ways in which Western blot results can be interpreted [36]. In the present study criteria were used that were established in Europe because European *B. burgdorferi *strains are different from strains in the USA. Also the antibody response of European human patients with Lyme Borreliosis was found to be variable and more restricted than that in U.S. patients [37,38]. Antibody response seemed stronger in seropositive Bernese Mountain Dogs compared to positive control dogs in this study as the reaction in the ELISA was stronger. Rather than differences in spirochete strains, in duration of the infection or in number of reinfections, differences in the immune response of the hosts are a possible explanation for this finding.

## Conclusion

In conclusion this study showed that Bernese Mountain Dogs more often had antibodies against *B. burgdorferi *compared to control dogs. Breed predisposition for antibodies against *B. burgdorferi *has not been reported before. More investigations are needed to evaluate the biological reasons and consequences of infections with *B. burgdorferi *in Bernese Mountain Dogs.

## Methods

### Samples and dogs

The dogs whose owners belonged to the Club for Bernese Mountain Dogs in Switzerland were defined as the population to be examined. The number of dogs needed to predict the prevalence of antibodies in this population was calculated using the statistical software EpiInfo 6.1 (WHO, Genf, 1997). At least 131 Bernese Mountain Dogs were needed to predict an estimated prevalence of 10% with an accuracy of 5%. The prevalence of 10% was estimated according to prevalences found in the literature.

The minimum number of control dogs needed was calculated using the software WinEpiscope 2.0 (Nacho de Blas, Zaragossa, Spain, available online). The control dogs were to be long haired, large breed dogs resembling the Bernese Mountain Dogs in size and hair coats. The hair coat color of the control dogs was classified either as dark (similar to Bernese Mountain Dogs) or as fair.

Owners were contacted and volunteered to join the study after a call from the Swiss Club for Bernese Mountain Dogs and the Swiss Newfoundland and Landseer club. Others were directly contacted if it was known that they owned a dog eligible for the study.

Samples were collected between July 2002 and April 2003.

Dogs were included in the study if they were older than 4 months. The dogs were healthy according to the owners with no obvious signs of a specific disease evaluated by a complete blood count, a serum biochemical analysis and urinalysis. Serum biochemical analysis included determination of bilirubin, glucose, urea, creatinine, total protein, albumin, cholesterol, sodium, potassium, chloride, calcium and phosphorus concentrations and measurement of the activity of alkaline phosphatase, alanine transferase, aspartate transferase and amylase. Urinalysis included urine test strip (Combur-Test^®^, Roche Diagnostics GmbH, Mannheim Germany), microscopic examination of urine sediment and determination of urine specific gravity and urine protein:creatinine ratio. The geographical area of Switzerland where the dogs originated was not previously determined and depended on the place where owners who wanted to join the study lived. Each owner was asked to complete a questionnaire and give information about the health status of the dog (Table 2). One specific question was whether the dog often had attached ticks. However the category "often" was not further specified.

The environment in which the dogs lived was investigated in retrospect by telephone interview (Table 3).

### Serologic testing

An enzyme-linked immunosorbent assay (ELISA) for the detection of antibodies against *B. burgdorferi sensu lato *was performed in all dogs according to a method described earlier [39]. Briefly a whole cell sonicate of *B. burgdorferi sensu stricto *reference strain B31 (ATCC 35210) was used as antigen. The samples were previously absorbed with a heterologous sorbant consisting of washed formalin inactivated whole cells of *E. coli*, *Salmonella typhimurium*, *Brachispira hyodysenteriae*, *Bacillus subtilis *and leptospires comprising 18 serovars. Western blot examinations for the detection of antibodies against *B. burgdorferi *were performed in all but one dog. A commercial test kit adapted for dogs was used (Virion Ltd., Rüschlikon, Switzerland). The tests which were performed according to the manufacturer's instructions consisted of Western blot strips with defined partial antigens of *B. burgdorferi ss. *and *B. afzelii*. In preliminary tests with positive and negative dog serum, a dilution of 1:200 and a conjugate (alkaline-phosphatase-rabbit-anti-dog IgG, H+L, Sigma, Diesenhofen, Switzerland) dilution of 1: 2000 was considered adequate. The interpretation of the Western blot results was done according to the interpretation criteria recommended for three European species of *B. burgdorferi sensu lato *[37]. Samples were considered positive if bands at the level of the partial antigens p100, p58, OspC, p21 or wb18 were identified or if at least two bands at the level of the partial antigens p45, bmpa und wb30 were present. Bands at the level of the partial antigens OspB, OspA, OspD, wb22 und OspE were considered unspecific.

For the microscopic agglutination test (MAT) to detect antibodies against leptospires, the ten most commonly recognized serovars (sv.) in Switzerland were used as antigens: *Leptospira interrogans*, sv.: *australis*, *bratislava*, *autumnalis*, *bataviae*, *canicola*, *grippotyphosa*, *icterohaemorrhagiae *and *pomona*; *Leptospira borgpetersenii*, sv.: *hardjo *and *tarassovi*.

### Statistical analysis

Data were recorded and analyzed using a commercial computer program (Statistical Package for the Social Sciences for Windows version 11, SPSS Inc., Chicago Il, USA). Between Bernese Mountain Dogs and control dogs variables were compared using the Mann-Whitney U test for the evaluation of age, optical density, percentage of time spent in the woods and a Fisher's exact test for all other data. Differences were considered significant at p < 0.05.

## Authors' contributions

BG: Designed the study, analyzed the data and drafted the manuscript.

SE: Contributed to the study design, collected the data and contributed to the manuscript drafting and data interpretation.

MMW: Performed the serologic tests, was involved in the study design and the drafting of the manuscript

CER: Was involved in the study design and coordination and contributed to the critical evaluation and interpretation of the data.

## References

[B1] Dambach DM, Smith CA, Lewis RM, Van Winkle TJ (1997). Morphologic, immunohistochemical, and ultrastructural characterization of a distinctive renal lesion in dogs putatively associated with Borrelia burgdorferi infection: 49 cases (1987–1992). Vet Pathol.

[B2] Grauer GF, Burgess EC, Cooley AJ, Hagee JH (1988). Renal lesions associated with Borrelia burgdorferi infection in a dog. J Am Vet Med Assoc.

[B3] Magnarelli LA, Anderson JF, Schreier AB, Ficke CM (1987). Clinical and serologic studies of canine borreliosis. J Am Vet Med Assoc.

[B4] Minkus G, Breuer W, Wanke R, Reusch C, Leuterer G, Brem G, Hermanns W (1994). Familial nephropathy in Bernese mountain dogs. Vet Pathol.

[B5] Reusch C, Hoerauf A, Lechner J, Kirsch M, Leuterer G, Minkus G, Brem G (1994). A new familial glomerulonephropathy in Bernese mountain dogs. Vet Rec.

[B6] Houston DM, Ribble CS, Head LL (1996). Risk factors associated with parvovirus enteritis in dogs: 283 cases (1982–1991). J Am Vet Med Assoc.

[B7] Arnold S, Müller A, Binder H, Meyers K, Giger U (1995). Von Willebrand factor concentrations in blood plasma of Bernese mountain dogs. Schweiz Arch Tierheilkd.

[B8] Kraus KH, Johnson GS, Kirk RW, Bonagura JD (1989). Von Willebrand's disease in dogs. Current Veterinary Therapy X.

[B9] Kathmann I, Jaggy A, Busato A, Bartschi M, Gaillard C (1999). Clinical and genetic investigations of idiopathic epilepsy in the Bernese mountain dog. J Smal Anim Pract.

[B10] Padgett GA, Madewell BR, Keller ET, Jodar L, Packard M (1995). Inheritance of histiocytosis in Bernese mountain dogs. J Small Anim Pract.

[B11] Voegeli E, Welle M, Hauser B, Dolf G, Fluckiger M (2006). Histiocytic sarcoma in the Swiss population of Bernese mountain dogs: a retrospective study of its genetic predisposition. Schweiz Arch Tierheilkd.

[B12] Watson PJ, Wotton P, Eastwood J, Swift ST, Jones B, Day MJ (2006). Immunoglobulin deficiency in Cavalier King Charles Spaniels with Pneumocystis pneumonia. J Vet Intern Med.

[B13] Cohen ND, Carter CN, Thomas MA, Angulo AB, Eugster AK (1990). Clinical and epizootiologic characteristics of dogs seropositive for Borrelia burgdorferi in Texas: 110 cases (1988). J Am Vet Med Assoc.

[B14] Alitalo A, Meri T, Ramo L, Jokiranta TS, Heikkila T, Seppala IJ, Oksi J, Viljanen M, Meri S (2001). Complement evasion by *Borrelia burgdorferi*: serum-resistant strains promote C3b inactivation. Infect Immun.

[B15] Wormser GP, Kaslow R, Tang J, Wade K, Liveris D, Schwartz I, Klempner M (2005). Association between human leukocyte antigen class II alleles and genotype of Borrelia burgdorferi in patients with early lime disease. J Infect Dis.

[B16] Yunis EJ, Larsen CE, Fernandez-Vina M, Awdeh ZL, Romero T, Hansen JA, Alper CA (2003). Inheritable variable sizes of DNA stretches in human MHC: conserved extended haplotypes and their fragments or blocks. Tissue Antigens.

[B17] Sjöwall J, Carlsson A, Vaarala O, Bergstrom S, Ernerudh J, Forsberg P, Ekerfelt C (2005). Innate immune responses in Lyme borreliosis: enhanced tumor necrosis factor-alpha and interleukin-12 in asymptomatic individuals in response to live spirochetes. Clin Exp Immunol.

[B18] Blum JR, Cork LC, Morris JM, Olson JL, Winkelstein JA (1985). The clinical manifestation of a genetically determined deficiency of the third component of complement in the dog. Clin Immunol Immunop.

[B19] Aeschlimann A, Chamot E, Gigon F, Jeanneret JP, Kesseler D, Walther Ch (1986). B. burgdorferi in Switzerland. Zbl Bakt Hyg A.

[B20] Jouda F, Crippa M, Perret J, Gern L (2003). Distribution and prevalence of Borrelia burgdorferi sensu lato in Ixodes ricinus ticks of canton Ticino (Switzerland). Eur J Epidemiol.

[B21] Jouda F, Perret J, Gern L (2004). Density of questing Ixodes ricinus nymphs and adults infected by Borrelia burgdorferi sensu lato in Switzerland: spatio-temporal pattern at a regional scale. Vector Borne Zoonotic Dis.

[B22] Jouda F, Perret J-L, Gern L (2004). Ixodes ricinus density, and distribution and prevalence of Borrelia burgdorferi sensu lato infection along an altitude gradient. J Med Entomol.

[B23] Miserez V, Gern L, Aeschlimann A (1990). Borrelia burgdorferi in ticks of the canton Tessin (Switzerland). Parasitologia.

[B24] Péter O, Bretz A-G, Bee D (1995). Occurrence of different genospecies of Borrelia burgdorferi sensu lato in ixodid ticks of Valais, Switzerland. Eur J Epidemiol.

[B25] Wicki R, Sauter P, Mettler C, Natsch A, Enzler T, Pusterla N, Kuhnert P, Egli G, Bernasconi M, Lienhard R, Lutz H, Leutenegger CM (2000). Swiss Army survey in Switzerland to determine prevalence of Francisella tularensis, members of the Ehrlichia phagocytophila genogroup, Borrelia burgdorferi sensu lato, and tick-borne encephalitis virus in ticks. Eur J Clin Microbiol.

[B26] Goossens HAT, van den Bogaard AE, Nohlmans MKE (2001). Dogs as sentinels for human Lyme borreliosis in the Netherlands. J Clin Microbiol.

[B27] Guerra M, Walker E, Kitron U (2001). Canine surveillance system for Lyme borreliosis in Wisconsin and Northern Illinois: Geographic distribution and risk factor analysis. Am J Trop Med Hyg.

[B28] Crippa M, Rais O, Gern L (2002). Investigations on the mode and dynamics of transmission and infectivity of Borrelia burgdorferi sensu stricto and Borrelia afzelii in Ixodes ricinus ticks. Vector Borne Zoonotic Dis.

[B29] DeSilva AM, Telford SR, Brunet LR, Barthold SW, Fikrig E (1996). Borrelia burgdorferi OspA is an arthropod-specific transmission-blocking Lyme disease vaccine. J Exp Med.

[B30] Shih CM, Pollack RJ, Telford SR, Spielman A (1993). Delayed dissemination of Lyme disease spirochetes from the site of deposition in the skin of mice. J Infect Dis.

[B31] Stjernberg L, Berglund J (2005). Detecting ticks on light versus dark clothing. Scand J Infect Dis.

[B32] Magnarelli LA, Ijdo JW, Padula SJ, Flavell RA, Fikrik E (2000). Serologic diagnosis of Lyme borreliosis by using enzyme-linked immunosorbent assays with recombinant antigens. J Clin Microbiol.

[B33] Shin SJ, Chang YF, Jacobson RH, Shaw E, Lauderdale TL, Appel MJ, Lein DH (1993). Cross-reactivity between B. burgdorferi and other spirochetes affects specificity of serotests for detection of antibodies to the Lyme disease agent in dogs. Clin Microbiol.

[B34] Steger-Lieb A, Gerber B, Nicolet J, Gaschen F (1999). An old disease with a new face: canine leptospirosis does not lose its relevance. Schweiz Arch Tierheilkd.

[B35] Guerra M, Walker E, Kitron U (2000). Quantitative approach for the serodiagnosis of canine Lyme disease by the Immunoblot procedure. J Clin Microbiol.

[B36] Littman MP (2003). Canine borreliosis. Vet Clin N Am Small Anim Pract.

[B37] Hauser U, Lehnert G, Lobentanzer R, Wilske B (1997). Interpretation criteria for standardized western blots for three European species of Borrelia burgdorferi sensu lato. J Clin Microbiol.

[B38] Dressler F, Ackermann R, Steere AC (1994). Antibody responses to the three genomic groups of Borrelia burgdorferi in European Lyme borreliosis. J Infect Dis.

[B39] Wittenbrink M, Failing K, Krauss H (1996). Enzyme-linked immunosorbent assay and immunoblot analysis for detection of antibodies to Borrelia burgdorferi in dogs. The impact of serum absorption with homologous and heterologous bacteria. Vet Microbiol.

